# Personality traits and physical activity in patients with gambling disorder attending a rehabilitation center. An observational study

**DOI:** 10.3389/fpsyg.2024.1465195

**Published:** 2024-11-12

**Authors:** Inmaculada Fierro, Raúl Fernández-Prieto, Alicia Fernández-Parra, Miriam Herrero-Martín, Azael J. Herrero

**Affiliations:** ^1^Department of Health Sciences, European University Miguel de Cervantes, Valladolid, Spain; ^2^Faculty of Education and Social Work, University of Valladolid, Valladolid, Spain; ^3^Research Center on Physical Disability, ASPAYM Castilla y León, Valladolid, Spain

**Keywords:** addictive behavior, behavior therapy, lifestyle, logistic regression, pathological gambling, personality disorders, sedentary behavior, self destructive behavior

## Abstract

**Objective:**

Problem gambling is associated with various negative health behaviors, such as physical inactivity. However, physical activity may also be used as a coping mechanism to manage stress and anxiety. This study aimed to investigate whether personality traits are associated with physical activity levels in individuals attending a rehabilitation center for gambling disorder (GD).

**Methods:**

An observational study was conducted in 71 patients belonging to a Gamblers’ Recovery Association. All of them completed a socio-demographic questionnaire, the Exploratory Personality Questionnaire-III (CEPER-III) and the International Physical Activity Questionnaire (IPAQ). Comparisons with general population and association between personality traits and physical activity were analyzed.

**Results:**

The study sample predominantly consisted of male participants (91.5%), with the majority having an educational attainment of compulsory schooling or less (70.4%). Additionally, a substantial proportion of participants exhibited school-related problems (43.7%) and had a history of mental health issues (33.8%). Compared to the general population, individuals in the CEPER-III cohort demonstrated significantly lower scores in the following personality traits: paranoid (*p* < 0.05), histrionic (*p* < 0.001), narcissistic (*p* < 0.001), passive-aggressive (*p* < 0.05), and sadistic (*p* < 0.01). Multivariate logistic regression analysis indicated that the antisocial, borderline, obsessive-compulsive, and self-destructive personality traits were significantly associated (*p* < 0.05) with the level of physical activity.

**Conclusion:**

This study found a link between personality traits and physical activity levels in patients with GD. Gamblers with higher scores on obsessive-compulsive and self-destructive personality traits were more likely to fall into the moderate-high physical activity group. In contrast, those with higher scores on antisocial and borderline personality traits were more likely to be classified in the low physical activity group.

## 1 Introduction

According to the American Psychiatric Association (APA), pathological gambling is defined as “a persistent and maladaptive gambling behavior that generates clinically significant distress.” In the latest version of the Diagnostic and Statistical Manual of Mental Disorders (DSM-5), pathological gambling was renamed as gambling disorder (GD) and moved from impulse control disorders to the section of behavioral addiction in the chapter of “Substance-Related and Addictive Disorders” (American Psychiatric Association, 2013; Potenza et al., 2019). Problem gambling is a public health issue that causes harm at both the individual and societal levels, impacting physical health and leading to negative health behaviors.

Whereas physical activity has emerged as a fundamental component of contemporary public health initiatives, problem gambling is associated with various negative health behaviors (Butler et al., 2020; Erickson et al., 2005). Individuals exhibiting heightened gambling preoccupation could spend significantly increased time engaged in sedentary behaviors, such as prolonged sitting with gambling-related activities. This temporal displacement of potential physical activity opportunities may contribute to elevated risks of sedentary lifestyle and its associated health consequences (Algren et al., 2015; Okechukwu, 2019). Physical inactivity is now recognized as the fourth leading risk factor for global mortality (World Health Organization, 2010).

The relationship between problem gambling and physical activity is not entirely straightforward and can vary among individuals. Problem gambling often co-occurs with mental health issues such as depression, anxiety and stress, that may contribute to decreased motivation for physical activity (Blaszczynski and Steel, 1998; Cunningham-Williams et al., 1998; Dowling et al., 2015; Kruedelbach et al., 2006; Wullinger et al., 2023). However, while some people avoid physical activity as a maladaptive way to cope with gambling problems, others may use physical activity as a coping mechanism to manage stress, anxiety, or other negative emotions. Exercise can provide a healthy outlet to relieve stress and ward off gambling-related thoughts and impulses, as well as promote cardiovascular health and the prevention of chronic diseases associated with a sedentary lifestyle (Okechukwu, 2019).

Research on this topic has produced mixed findings, with some studies suggesting that problem gamblers may be less physically active than the general population, while others have found no significant differences or even higher levels of physical activity among problem gamblers (de Leeuw et al., 2010; Kwok et al., 2021; Yamada et al., 2023). On the other hand, sociodemographic factors such as age, sex, socioeconomic level, etc., can influence an individual’s level of physical activity regardless of whether they have gambling problems or not (Algren et al., 2015; Brodersen et al., 2005; Ortiz-Hernández and Ramos-Ibáñez, 2010). Additionally, problem gambling can lead to financial difficulties, which can limit people’s access to resources for physical activity. High levels of debt and financial stress can restrict participation in activities requiring financial investment, such as gym memberships, sports teams, or recreational classes (Langham et al., 2015; Wilson et al., 2024).

Another factor to consider with patients with GD is how the stages of addiction can impact activity levels. During active gambling, individuals might neglect exercise due to preoccupation with gambling. Alternatively, they might engage in high-risk activities as part of their addiction. During recovery, individuals might intentionally use exercise to manage cravings and improve well-being (Angelo et al., 2013).

Personality traits can play a significant role in influencing individuals’ engagement in physical activity (Rhodes and Smith, 2006; Rhodes and Boudreau, 2017). On the other hand, research suggests that individuals with GD may exhibit distinct personality traits compared to the general population or non-pathological gamblers (Kaur et al., 2023; MacLaren et al., 2011; Myrseth et al., 2016). The relationship between personality traits and the level of physical activity has been analyzed in the general population finding small positive associations between extraversion and conscientiousness and physical activity, and a small negative relationship with neuroticism. Likewise, the associations with openness to experience and agreeableness are generally trivial. These findings are largely consistent across different demographic characteristics and study designs, suggesting that the relationship between personality and physical activity is relatively stable (Allen and Laborde, 2014; Rhodes and Boudreau, 2017). However, the influence of personality traits on problem gamblers’ physical activity is less known. Therefore, this study aimed to investigate whether personality traits are associated with physical activity levels in individuals attending a rehabilitation center for GD. Taking into account the correlation between the personality traits of the Big Five and the CEPER III (Caballo et al., 2009), we hypothesized that individuals with GD who exhibit higher levels of neuroticism (borderline, passive-aggressive & depressive) and lower levels of extraversion (avoidant) and conscientiousness (obsessive- compulsive) will have lower levels of physical activity.

## 2 Materials and methods

### 2.1 Design and participants

**Figure 1:**
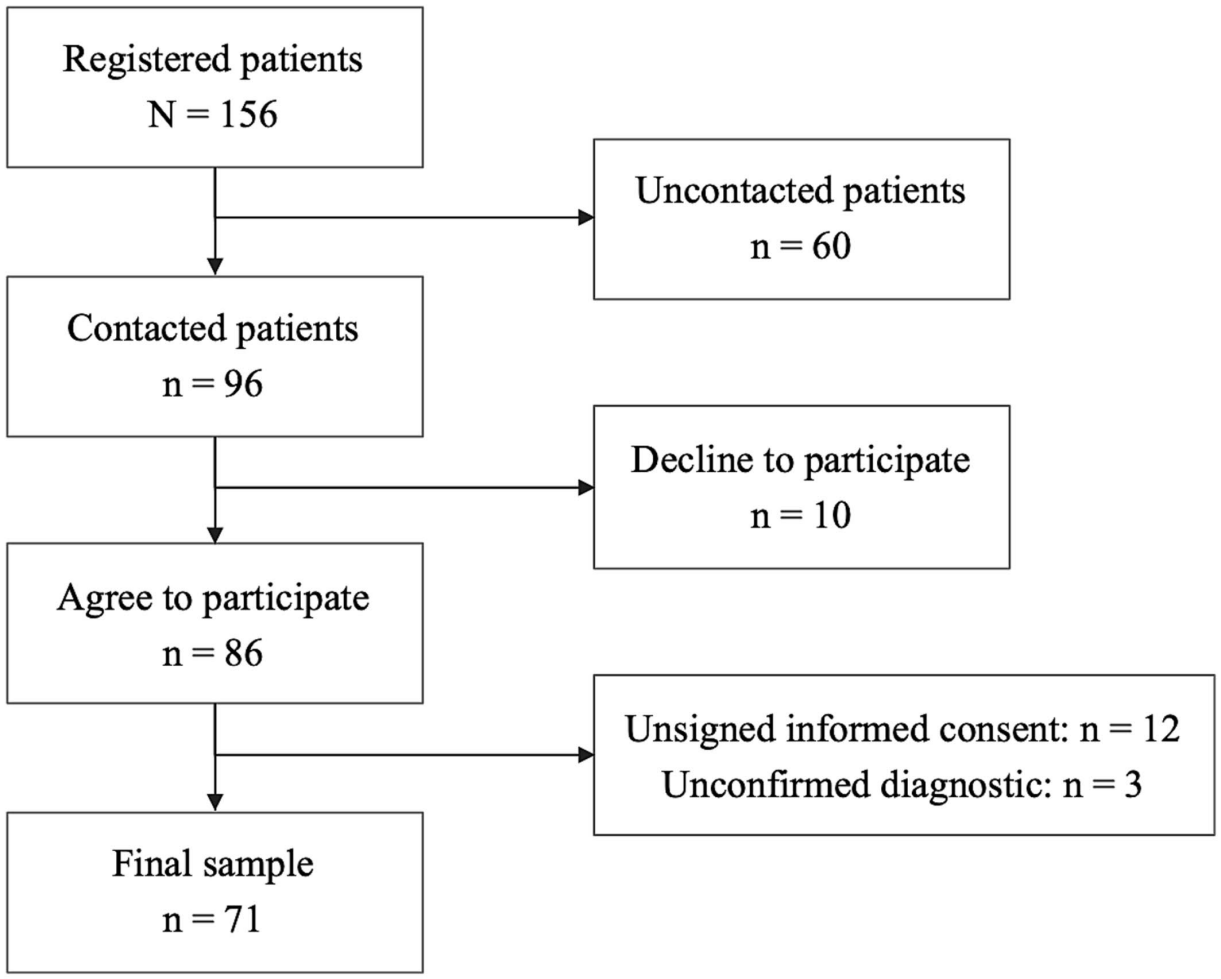
Flowchart for the inclusion of patients in the study.

An observational study was conducted in patients diagnosed with GD according to DSM-5 diagnostic criteria (American Psychiatric Association, 2013). The inclusion criteria were as follows: Adults (aged 18 years or older) with GD diagnosis were recruited from the Gamblers’ Recovery Association (AJUPAREVA, Valladolid, Spain). Out of 156 patients registered in AJUPAREVA, only 96 were successfully contacted, of which 10 declined to participate in the study. Of the 86 who agreed to participate, 12 were excluded for not signing the informed consent form and two for lack of confirmation of GD diagnosis. The final sample size was 71 patients. Figure 1 shows the flowchart for the inclusion of participants in the study. After agreeing to participate in the study, all respondents completed the CEPER-III and IPAQ tests.

Both the socio-demographic variables and the clinical background of the study participants were incorporated into a database based on the patients’ responses to the intake interview at the association. The results of the “Exploratory Personality Questionnaire-III” (CEPER-III) and the “International Physical Activity Questionnaire” (IPAQ) questionnaires were added to this database. Both questionnaires were administered by a collaborating psychologist from the AJUPAREVA association. Researchers received an anonymized database file in Excel format.

### 2.2 Instruments

#### 2.2.1 Socio-demographic characteristics

In addition to the main study variables (personality traits and physical activity levels), the variables listed in Table 1 were considered, based on the patients’ responses to the admission interview at the association, as indicated above. These variables were: sex, age, marital status, educational level, occupation, type of gambling dependence, consumption episodes, withdrawal syndrome, time elapsed from the patient’s awareness of their GD to seeking help at the center, global loss estimation, family finances, school problems and mental health history.

**Table 1:** Characteristics of the sample.

	*n* (%)
Sociodemographic characteristics	
Sex	
Female	6 (8.5)
Male	65 (91.5)
Age (y/o)	
20–30	18 (25.4)
31–40	17 (23.9)
41–50	19 (26.8)
> 50	17 (23.9)
Marital status	
Married/ cohabiting	32 (45.1)
Single	34 (47.9)
Separated/ divorced/ widow	4 (5.6)
Unknown	1 (1.4)
Educational level	
Compulsory schooling or less	50 (70.4)
High or vocational school	13 (18.3)
College/ university	7 (9.9)
Other	1 (1.4)
Occupation	
Entry-level	10 (14.1)
Mid-level	25 (35.2)
Student	5 (7.0)
Other	31 (43.7)
Background for gambling behavior, clinical and family characteristics	
Type of gambling dependence	
Land-based gambling	50 (70.4)
Online gambling	18 (25.4)
Unknown	3 (4.2)
Consumption episodes	
Almost daily	35 (49.3)
Weekly	5 (7.0)
Biweekly	4 (5.6)
Monthly	4 (5.6)
Sporadic	16 (22.5)
Unknown	7 (9.9)
Withdrawal syndrome (yes)	25 (35.2)
Time from awareness to admission (years)	4.00 [2.00–9.50]*
Estimation of overall losses in euros (thousands of euros)	25.00 [12.00–60.00]*
Family finances	
Normal	60 (84.5)
Material deprivation	6 (8.5)
Overabundance in media or availability	4 (5.6)
Unknown	1 (1.4)
School problems (yes)	31 (43.7)
Mental health history (yes)	24 (33.8)

^*^Median [Q_1_-Q_3_].

#### 2.2.2 Personality traits

The “Exploratory Personality Questionnaire-III” (CEPER-III) is a 170-item measure of 14 domains of personality: paranoid, schizoid, schizotypal, histrionic, narcissistic, antisocial, borderline, avoidant, dependent, obsessive compulsive, passive aggressive, sadistic, self-destructive and depressive. Additionally, two items are included that evaluate sincerity and that attempt to rule out the possibility that the subjects answer the questionnaire at random. The instrument was built and validated in Spain as an alternative to measure personality styles in a dimensional way. Internal consistency of the scales was assessed using Cronbach’s alpha, which ranged from 0.75 to 0.89, with an overall alpha for the test of 0.97 (Caballo et al., 2011). The complete questionnaire is available in the article by Caballo et al. (2011). Its convergent validity was obtained by comparing it with the “Millon Clinical Multiaxial Inventory” (Millon, 2006).

#### 2.2.3 Physical activity

The “International Physical Activity Questionnaire” (IPAQ) emerged as a collaborative effort among researchers from various countries and academic institutions with the aim of developing a standardized instrument for assessing physical activity levels across diverse populations. The initiative stemmed from the growing recognition of the importance of physical activity in promoting health and preventing chronic diseases, coupled with the need for a reliable and valid tool to measure physical activity on a global scale.

IPAQ researchers developed several versions of the instrument according to the number of questions (short or long), the recall period (“usually in 1 week” or “last 7 days”) and the method of application (self-administered survey, face-to-face interview or by telephone). The questionnaires were designed to be used in adults aged 18 to 65 years. We used the short version, which consists of 9 items and provides information on the time spent walking, in moderate and vigorous intensity activities and in sedentary activities (Supplementary material S1). Different studies suggest that the short version is the one used in population studies (Kurita et al., 2021; Oliveira et al., 2023).

### 2.3 Statistical analysis

For qualitative variables (sex, age -in ranges-, marital status, educational level, occupation, type of gambling dependence, consumption episodes, withdrawal syndrome, family finances, school problems, mental health history and physical activity level), absolute frequencies and percentages are presented, and for quantitative variables (time from awareness to admission -in years-, estimation of overall losses -in thousands of euros- and personality traits scores -in points-), the mean and standard deviation (SD) or the median and interquartile range [Q1-Q3] are provided. The normality of quantitative variables was analyzed using the Komogorov-Smirnov or Shapiro–Wilk goodness-of-fit tests, depending on the group size (n > 50 or n ≤ 50, respectively). We used a one-sample T-test to determine whether the means of pathological gamblers’ personality traits in our sample differed significantly from the mean reported in the study by Caballo et al. (2011) for the Spanish population. Internal consistency of the scales as well as of overall test was assessed using Cronbach’s alpha. Hypothesis testing was used to analyze the potential association between personality traits and the level of physical activity. Pearson’s chi-square test of independence (χ^2^) was used for group comparisons when both variables were qualitative, and the independent T-Student test or the Mann–Whitney U test were used for independent two groups and one-way ANOVA or Kruskal-Wallis’s test for three groups when the variable was quantitative. Multivariate logistic regression was the method used to analyze sociodemographic and personality traits characteristics associated with the level of physical activity. Odds ratios with their 95% confidence interval (OR [95% CI]) are presented as measures of the magnitude of the association between the main factors analyzed and the level of physical activity (moderate-high /low). The possible multicollinearity of the model was checked with an analysis of tolerance and inflation statistics. To ensure model reliability, we assessed calibration with the Hosmer-Lemeshow test and evaluated discrimination ability using the area under the curve (AUC) of the ROC (Receiver Operating Characteristic) curve. Internal validation of the logistic regression model employed simple bootstrapping with 1,000 resamples. For all tests, the significance level was set at *p* < 0.05. Statistical analysis was conducted using SPSS software v. 27.

## 3 Results

### 3.1 Socio-demographic characteristics and, background for gambling behavior, clinical and family characteristics

Table 1 shows the main socio-demographic characteristics and the background for gambling behavior, clinical and family characteristics of the sample. No significant associations between the variables in Table 1 were found to be relevant to the purpose of this study.

### 3.2 Personality traits of pathological gamblers

Cronbach’s alpha for the CEPER-III subscales ranged from 0.81 to 0.92. The overall test yielded a coefficient of 0.98. These values indicate good to high internal consistency, suggesting that the CEPER-III subscales and the total score measure a unitary latent construct reliably.

Table 2 shows, for men and women, means scores for personality traits from the current study and for the study of Caballo et al. (2011). Patients with GD in rehabilitation exhibited lower scores than general population on paranoid, histrionic, narcissistic, passive-aggressive, and sadistic personality in men and histrionic personality trait in women.

**Table 2:** CEPER-III personality traits scores in patients with GD vs. Spanish general population*.

Personality trait	Men				Women			
	Pathological gamblers (*n* = 58) Mean (SD)	Spanish general population* (*n* = 197) Mean	*t*	*p*	Pathological gamblers (*n* = 6) Mean (SD)	Spanish general population* (*n* = 304) Mean	*t*	*p*
Paranoid	28.76 (11.99)	32.73	−2.522	**0.014**	31.83 (10.40)	29.83	0.472	0.657
Schizoid	30.76 (11.18)	31.16	−0.273	0.786	34.50 (9.85)	27.5	1.740	0.142
Schizotypal	22.29 (9.14)	24.3	−1.673	0.100	25.00 (11.82)	23.04	0.406	0.701
Antisocial	26.83 (10.64)	28.96	−1.526	0.132	25.17 (12.42)	25.71	−0.107	0.919
Borderline	28.48 (11.63)	28.45	0.021	0.983	36.83 (11.20)	28.86	1.744	0.142
Histrionic	34.45 (10.52)	39.55	−3.693	**<0.001**	28.17 (8.18)	39.03	−3.252	**0.023**
Narcissistic	33.36 (11.79)	38.42	−3.268	**0.002**	25.33 (9.31)	34.92	−2.522	0.053
Avoidant	30.67 (14.45)	31.59	−0.484	0.630	39.67 (9.56)	31.57	2.074	0.093
Dependent	32.88 (11.63)	33.92	−0.681	0.498	41.67 (9.03)	35.86	1.576	0.176
Obsessive- compulsive	43.55 (12.98)	42.54	0.593	0.555	38.67 (9.07)	41.4	−0.738	0.494
Passive-aggressive	30.50 (10.91)	34.16	−2.556	**0.013**	33.00 (15.01)	31.79	0.198	0.851
Self-destructive	26.12 (9.83)	27.83	−1.325	0.191	32.17 (7.78)	26.35	1.831	0.127
Depressive	31.95 (14.89)	28.72	1.651	0.104	38.83 (10.57)	29.36	2.195	0.080
Sadistic	17.64 (7.16)	20.59	−3.141	**0.003**	19.33 (7.69)	17.47	0.594	0.578

^*^Data from Caballo et al. (2011). Differences have been highlighted in bold.

### 3.3 Physical activity in pathological gamblers

Table 3 shows the scores for the personality traits obtained by the CEPER-III in the sample, grouping it by the level of physical activity obtained by the IPAQ. Those people with moderate and high activity levels have been grouped in a fourth column. No significant differences were observed in personality traits depending on the level of physical activity, neither when grouped into three groups (low vs. moderate vs. high; one-way ANOVA or Kruskal-Wallis’s test) nor into two groups (low vs. moderate or high; T-Student test or the Mann–Whitney U test), except for obsessive-compulsive trait (*p* < 0.05).

**Table 3:** Mean scores for personality traits according to the CEPER-III by level of physical activity IPAQ.

Personality trait	Low (*n* = 10)	Moderate (*n* = 27)	High (*n* = 25)	Moderate or high (*n* = 52)
	Mean (SD)	Mean (SD)	Mean (SD)	Mean (SD)
Paranoid	24.40 (15.37)	29.44 (10.66)	30.40 (11.54)	29.90 (10.99)
Schizoid	26.40 (13.08)	32.89 (9.17)	31.12 (11.81)	32.04 (10.45)
Schizotypal	21.40 (10.48)	22.85 (8.62)	22.52 (9.66)	22.69 (9.050)
Antisocial	29.30 (14.77)	25.30 (9.39)	27.00 (10.24)	26.12 (9.75)
Borderline	32.10 (16.2)	29.30 (9.33)	27.76 (12.34)	28.56 (10.80)
Histrionic	30.60 (13.37)	34.00 (9.86)	35.12 (10.33)	34.54 (10.01)
Narcissistic	29.10 (14.37)	33.11 (11.56)	34.32 (11.00)	33.69 (11.20)
Avoidant	33.50 (20.56)	31.93 (12.88)	30.16 (13.12)	31.08 (12.90)
Dependent	33.20 (15.27)	34.81 (11.12)	32.44 (10.68)	33.67 (10.87)
Obsessive- compulsive	33.80 (16.69)	44.19 (10.93)	46.04 (11.63)*	45.08 (11.20)**
Passive-aggressive	30.00 (13.05)	30.37 (10.51)	30.76 (10.83)	30.56 (10.56)
Self-destructive	23.30 (9.89)	27.44 (7.65)	27.36 (11.65)	27.40 (9.68)
Depressive	34.40 (17.47)	32.93 (13.80)	31.48 (15.29)	32.23 (14.41)
Sadistic	15.40 (7.09)	18.37 (5.99)	18.28 (8.50)	18.33 (7.23)

* and ** significantly different from low level with *p* < 0.05 and *p* < 0.01, respectively.

### 3.4 Personality traits and physical activity in patients with GD

Multivariate logistic regression analysis (Table 4) revealed that certain personality traits are associated with the level of physical activity in patients attending a rehabilitation center for GD. Using patients with “moderate or high levels” of physical activity as the reference group, the probability of belonging to the “low” physical activity group increases with increasing scores on the “antisocial” and “borderline” personality traits and decreases with increasing scores on the “obsessive-compulsive” and “self-destructive” traits.

**Table 4:** OR for personality traits associated with “low level” of physical activity (*vs* “moderate or high levels”) and adjusted OR by multivariate logistic regression.

	Multivariate model	
Personality trait	OR [95% CI]	OR [95% CI]
Paranoid	0.95 [0.89–1.02]	
Schizoid	0.95 [0.88–1.02]	
Schizotypal	0.98 [0.91–1.06]	
Antisocial	1.03 [0.97–1.09]	1.16 [1.01–1.34]*
Borderline	1.03 [0.97–1.08]	1.19 [1.00–1.41]*
Histrionic	0.96 [0.90–1.03]	
Narcissistic	0.97 [0.91–1.03]	
Avoidant	1.01 [0.97–1.06]	
Dependent	1.00 [0.94–1.06]	
Obsessive- compulsive	0.92 [0.86–0.98]*	0.86 [0.77–0.97]*
Passive-aggressive	1.00 [0.93–1.06]	
Self-destructive	0.95 [0.87–1.03]	0.76 [0.62–0.94]*
Depressive	1.01 [0.97–1.06]	
Sadistic	0.92 [0.80–1.06]	

^*^p < 0.05.

**Figure 2:**
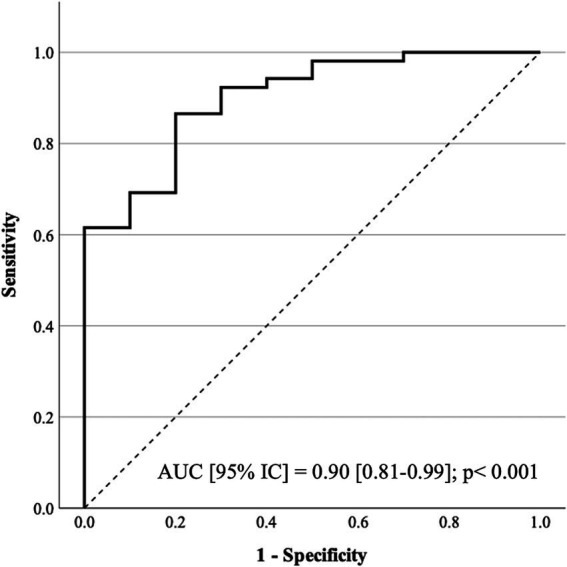
Receiver operating characteristic (ROC) curve: predicted probability of the logistic regression model for low physical activity.

The multivariate model (Table 4) exhibits good calibration (Hosmer and Lemeshow test: χ^2^ = 5.30; *p* = 0.73) and discrimination (Figure 2), with internal validation through bootstrapping (1,000 resamples) confirming the coefficient values and their significance.

## 4 Discussion

This study aimed to investigate whether personality traits are associated with physical activity levels in in patients with GD attending a rehabilitation center. The main findings of the present study showed that the level of physical activity varies with some problem gamblers’ personality traits. While elevated scores on the ‘obsessive-compulsive’ and ‘self-destructive’ trait scales increases the likelihood of being classified within the ‘moderate-high’ level of physical activity group, elevated scores on the ‘antisocial’ and ‘borderline’ personality traits enhance the likelihood of belonging to the ‘low’ physical activity group.

A significantly higher proportion of men than women came seeking treatment for GD. The higher prevalence of GD among men compared to women has been well-documented, with numerous studies exploring the underlying factors contributing to this gender disparity (Carneiro et al., 2020; Potenza et al., 2019; Welte et al., 2015; Wong et al., 2013). While biological, psychological, social, cultural and environmental factors have been implicated (Blanco et al., 2006; Fattore et al., 2014; Miller et al., 2023; Moreira et al., 2023; Potenza et al., 2019; Xuan et al., 2017), they cannot fully explain the observed disproportion in treatment-seeking behavior, also observed in other studies (Jiménez-Murcia et al., 2019). Some researchers suggest that female gamblers may face greater stigmatization and shame compared to their male counterparts, discouraging them from seeking professional help (Baxter et al., 2016; Grunfeld et al., 2004). This highlights the need to consider gender-specific factors when designing and implementing GD prevention and treatment programs.

The current study’s participants exhibited sociodemographic characteristics (male, over 35 years old, and basic education level) that align with findings from previous research on pathological gamblers in Spain (Sáez-Abad and Bertolín-Guillén, 2008). However, a striking observation was the relatively high proportion of young help-seekers: one-quarter of the participants fell within the 20–30 year-old age range. While various studies report a heterogeneous distribution of GD prevalence across lifespan, a potential peak prevalence is often observed during early to mid-adulthood (25–45 years old) (Chóliz et al., 2021; Moreira et al., 2023), probably linked to increased financial independence during these years. Considering the typical delay between problem gambling onset and seeking help, one might expect a lower representation of individuals in the 20–30 year old age group compared to older age ranges. This fact shows that rehabilitation programs for pathological gamblers should not be limited to adults in middle age only, but should also cover all age groups, including the youngest as our results show.

Low education, school problems, and psychiatric history were common among participants. This aligns with past research on pathological gamblers (Jiménez-Murcia et al., 2019; Moreira et al., 2023; Sáez-Abad and Bertolín-Guillén, 2008). In addition, prior research shows that several mental health and psychiatric conditions are linked to gambling problems’ development (Kessler et al., 2008). Other research suggests that psychopathology, particularly attention deficit hyperactivity disorder (ADHD), is a GD predictor (Potenza et al., 2019). No association emerged among these variables in our study, but a trend suggests possible type II error analyzing association between psychiatric history and school problems, since among participants with reported psychiatric problems, nearly 60% also had a history of school problems. Regarding self-reported gambling losses, it should be noted that they must be interpreted with caution, as previous research has shown that gamblers tend to overestimate their losses (Braverman et al., 2014).

Personality manifests itself in the form of traits not necessarily denoting pathology but given the correlation found between the Millon and CEPER-III questionnaires, it would be expected that individuals with a personality disorder can obtain high scores in the related CEPER-III subscale (Caballo et al., 2011; Caballo et al., 2009; Furnham, 2022; Millon, 2006). Patients seeking treatment at AJUPAREVA exhibited average personality trait scores, largely comparable to those of the general population. Curiously, there were a few personality traits in which AJUPAREVA patients showed lower mean scores than the general population. Low scores on the paranoid, histrionic, narcissistic, passive-aggressive, and sadistic personality traits could reflect a stable and balanced personality (Caballo et al., 2009). Through the perspective of the Big Five personality model, present findings suggest a potential link between treatment-seeking behavior and pathological gamblers’ personality traits. Results may specifically indicate an “Emotional stability” and predominance of the “Agreeableness” dimension coupled with a relative weakness in “Extraversion.” Individuals high in “Agreeableness” tend to be cooperative, compassionate, and sensitive to the needs of others (Caballo et al., 2009; John and Srivastava, 1999; Furnham, 2022). This could translate into a heightened awareness of interpersonal difficulties and a greater willingness to seek professional help when social or emotional problems arise. However, “Agreeableness” can also be associated with a tendency to be trusting and potentially susceptible to exploitation (Costa and McCrae, 2010). On the other hand, lower levels of “Extraversion” reflect a preference for solitude and a focus on internal experiences. While introversion can limit social interaction, it can also foster introspection and self-awareness, potentially leading individuals to recognize and address emotional challenges (Rocklin and Revelle, 1981). However, limited social networks associated with introversion could create barriers to help-seeking behavior in some cases. It is crucial to note that these personality characteristics may not be representative of pathological gamblers as a whole (Kaur et al., 2023; MacLaren et al., 2011) but rather of those who actively seek treatment.

In the bivariate analysis, a difference was only observed in the obsessive-compulsive trait between the low and high levels of physical activity; however, the multivariate analysis showed that high scores in ‘obsessive-compulsive’ and ‘self-destructive’ traits are linked to higher physical activity levels, while high scores in ‘antisocial’ and ‘borderline’ traits are associated with lower physical activity levels. Although there are numerous studies that analyze the relationship between personality traits or personality disorders and physical activity, this is the first study, to the authors’ knowledge, using CEPER-III and relating it to the physical activity level in pathological gamblers. While the relationship between personality traits and physical activity is complex and varies among individuals, personality can significantly influence on both starting and sticking with regular exercise, as well as on physical performance (Rhodes and Smith, 2006; Rhodes and Boudreau, 2017; Allen and Laborde, 2014). Exercise has been shown to improve mood, reduce stress, and enhance overall well-being, which may be particularly relevant for individuals struggling with emotional dysregulation (Okechukwu, 2019). Borderline personality disorder (BPD) is characterized by emotional instability, impulsivity, and difficulties in maintaining stable relationships (American Psychiatric Association, 2013). People with borderline traits may exhibit impulsive behaviors, which can manifest in both positive and negative ways regarding physical activity. Impulsivity may lead to spontaneous bursts of energy and motivation for exercise, but it can also result in erratic exercise patterns or engaging in high-risk activities without considering potential consequences. Some individuals with borderline traits may use exercise as a coping mechanism to regulate intense emotions or alleviate distress. For example, engaging in vigorous exercise may help reduce anxiety or anger feelings. Conversely, emotional instability may also contribute to periods of low motivation or fluctuations in exercise adherence, particularly during heightened emotional distress times (Rhodes and Boudreau, 2017).

Allen and Laborde’s (2014) investigation indicate that individuals exhibiting traits associated with impulsivity and emotional instability (such as those found in antisocial and borderline personality disorders) are less likely to participate in regular physical activity. This reluctance is attributed to a combination of low self-regulation, motivation, and adherence to structured activities. Furthermore, individuals with antisocial traits often display a diminished concern for health outcomes, which further undermines their intrinsic motivation to maintain an active lifestyle.

These findings align with the behavioral patterns characteristic of individuals with antisocial traits, which include a propensity for risk-taking and a reduced capacity for long-term planning. Both factors negatively impact regular exercise participation. Personality-driven behaviors create barriers to consistent physical activity, contributing to a sedentary lifestyle. Additionally, emotional instability and impulsivity, common in borderline personality disorder, have been shown to correlate with a decreased likelihood of engaging in consistent physical activity routines (Rhodes and Smith, 2006).

The influence of obsessive-compulsive and self-destructive traits on physical activity is likely to be complex and context dependent. While certain aspects of these traits may promote engagement in physical activity, other facets may hinder it or lead to maladaptive behaviors. Individuals with obsessive-compulsive traits may exhibit high levels of organization, discipline, and attention to detail. These characteristics could potentially translate into structured exercise routines and adherence to fitness goals. They may be diligent about tracking their progress, following exercise plans, and maintaining consistency in their physical activity habits. While self-destructive traits are generally associated with negative behaviors and outcomes, they may also manifest in certain forms of physical activity. Some individuals with self-destructive tendencies may engage in extreme or high-risk sports or activities as a way to cope with emotional pain or seek adrenaline rushes. In these cases, physical activity serves as a means of self-expression or a release valve for intense emotions (Bottoms et al., 2023; Rhodes and Boudreau, 2017; Tang et al., 2023).

More research is needed to better understand the associations between problem gambling and physical activity and to identify effective strategies for promoting healthy behaviors among individuals with gambling problems. Overall, understanding the relationship between personality traits and physical activity can help to inform personalized approaches to promoting and maintaining healthy exercise habits in problematic gamblers. By recognizing individual differences in personality, health professionals can tailor interventions and support strategies to better meet each patient’s needs and preferences. Personalized interventions addressing the unique needs and circumstances of problem gamblers may be beneficial in promoting overall well-being and reducing the negative consequences of gambling disorder.

## 5 Limitations

While the present study provides valuable preliminary insights into the relationship between physical activity and personality traits in pathological gamblers attending a rehabilitation center, several limitations must be acknowledged. First, the small sample size limits the generalizability of the findings and reduces the statistical power of the analyses. Future research should aim to include a larger and more diverse sample, considering variations in gender and socioeconomic background, to enhance the external validity of the results. Additionally, the sequential design of this study constrains the ability to observe changes in personality traits and physical activity levels over time, particularly during different stages of addiction and recovery. A longitudinal design that tracks participants over an extended period would offer a more nuanced understanding of the dynamic interactions between these factors throughout the addiction and recovery process. Finally, the study’s reliance on self-reported questionnaires introduces potential bias, such as under- or over-reporting due to recall bias or social desirability. To mitigate these biases, future research should consider employing mixed-method approaches, including objective measures of physical activity (e.g., wearable fitness trackers) to supplement self-reported data and improve the reliability of the findings.

## 6 Conclusion

This study found a link between personality traits and physical activity levels in problem gamblers. Pathological gamblers with higher scores on obsessive-compulsive and self-destructive personality traits were more likely to fall into the moderate-high physical activity group. In contrast, those with higher scores on antisocial and borderline personality traits were more likely to be classified in the low physical activity group. These results suggest that the design of physical exercise programs for pathological gamblers must consider their personality traits so that physical exercise contributes to the treatment of their addictive behavior.

## Data Availability

The datasets presented in this article are not readily available because the original contributions presented in the study are included in the article, further inquiries can be directed to the corresponding author. Requests to access the datasets should be directed to jaherrero@uemc.es.

## References

[ref1] Algren M. H. Ekholm O. Davidsen M. Larsen C. V. L. Juel K. (2015). Health behaviour and body mass index among problem gamblers: results from a nationwide survey. J. Gambl. Stud. 31, 547–556. doi: 10.1007/s10899-013-9437-y, PMID: 24390713

[ref2] Allen M. S. Laborde S. (2014). The role of personality in sport and physical activity. Curr. Dir. Psychol. Sci. 23, 460–465. doi: 10.1177/0963721414550705

[ref3] American Psychiatric Association (2013). Diagnostic and statistical manual of mental disorders (DSM-5). Fifth Edn. Washington, DC: American Psychiatric Association.

[ref4] Angelo D. L. Tavares H. Zilberman M. L. (2013). Evaluation of a physical activity program for pathological gamblers in treatment. J. Gambl. Stud. 29, 589–599. doi: 10.1007/s10899-012-9320-2, PMID: 22661334

[ref5] Baxter A. Salmon C. Dufresne K. Carasco-Lee A. Matheson F. I. (2016). Gender differences in felt stigma and barriers to help-seeking for problem gambling. Addict. Behav. Rep. 3, 1–8. doi: 10.1016/j.abrep.2015.10.001, PMID: 29531995 PMC5845950

[ref6] Blanco C. Hasin D. S. Petry N. Stinson F. S. Grant B. F. (2006). Sex differences in subclinical and DSM-IV pathological gambling: results from the National Epidemiologic Survey on alcohol and related conditions. Psychol. Med. 36:943. doi: 10.1017/S0033291706007410 16650342

[ref7] Blaszczynski A. Steel Z. (1998). Personality disorders among pathological gamblers. J. Gambl. Stud. 14, 51–71. doi: 10.1023/a:1023098525869 12766434

[ref8] Bottoms L. Prat Pons M. Fineberg N. A. Pellegrini L. Fox O. Wellsted D.. (2023). Effects of exercise on obsessive-compulsive disorder symptoms: a systematic review and meta-analysis. Int. J. Psychiatry Clin. Pract. 27, 232–242. doi: 10.1080/13651501.2022.2151474, PMID: 36541901

[ref9] Braverman J. Tom M. A. Shaffer H. J. (2014). Accuracy of self-reported versus actual online gambling wins and losses. Psychol. Assess. 26, 865–877. doi: 10.1037/a0036428, PMID: 24708074

[ref10] Brodersen N. H. Steptoe A. Williamson S. Wardle J. (2005). Sociodemographic, developmental, environmental, and psychological correlates of physical activity and sedentary behavior at age 11 to 12. Ann. Behav. Med. 29, 2–11. doi: 10.1207/s15324796abm2901_2 15677295

[ref11] Butler N. Quigg Z. Bates R. Sayle M. Ewart H. (2020). Gambling with your health: associations between gambling problem severity and health risk Behaviours, health and wellbeing. J. Gambl. Stud. 36, 527–538. doi: 10.1007/s10899-019-09902-8, PMID: 31705379 PMC7214382

[ref12] Caballo V. Guillén J. Salazar I. (2009). Estilos, rasgos y trastornos de la personalidad: interrelaciones y diferencias asociadas al sexo. Psico 40, 319–327.

[ref13] Caballo V. E. Guillén J. L. Salazar I. C. Irurtia M. J. (2011). Estilos y trastornos de personalidad: Características psicométricas del “Cuestionario Exploratorio de Personalidad-III” (CEPER-III). Behav. Psychol. 19, 277–302.

[ref14] Carneiro E. Tavares H. Sanches M. Pinsky I. Caetano R. Zaleski M.. (2020). Gender differences in gambling exposure and at-risk gambling behavior. J. Gambl. Stud. 36, 445–457. doi: 10.1007/s10899-019-09884-7, PMID: 31471835

[ref15] Chóliz M. Marcos M. Lázaro-Mateo J. (2021). The risk of online gambling: a study of gambling disorder prevalence rates in Spain. Int. J. Ment. Heal. Addict. 19, 404–417. doi: 10.1007/s11469-019-00067-4

[ref16] Costa P. T. McCrae R. R. (2010). “The five-factor model, five-factor theory, and interpersonal psychology” in Handbook of Interpersonal Psychology: Theory, Research, Assessment, and Therapeutic Interventions. eds. Horowitz L. M. Strack S. (New York, NY, USA: John Wiley & Sons, Inc), 91–104.

[ref17] Cunningham-Williams R. M. Cottler L. B. Compton W. M. 3rd Spitznagel E. L. (1998). Taking chances: problem gamblers and mental health disorders--results from the St. Louis epidemiologic catchment area study. Am. J. Public Health 88, 1093–1096. doi: 10.2105/ajph.88.7.1093, PMID: 9663161 PMC1508270

[ref18] de Leeuw J. R. J. de Bruijn M. de Weert-van Oene G. H. Schrijvers A. J. P. (2010). Internet and game behaviour at a secondary school and a newly developed health promotion programme: a prospective study. BMC Public Health 10, 1–8. doi: 10.1186/1471-2458-10-544, PMID: 20828394 PMC2944373

[ref19] Dowling N. A. Cowlishaw S. Jackson A. C. Merkouris S. S. Francis K. L. Christensen D. R. (2015). Prevalence of psychiatric co-morbidity in treatment-seeking problem gamblers: a systematic review and meta-analysis. Austr. New Zeal. J. Psychiatry 49, 519–539. doi: 10.1177/0004867415575774, PMID: 25735959 PMC4438101

[ref20] Erickson L. Molina C. A. Ladd G. T. Pietrzak R. H. Petry N. M. (2005). Problem and pathological gambling are associated with poorer mental and physical health in older adults. Int. J. Geriatr. Psychiatry 20, 754–759. doi: 10.1002/gps.1357, PMID: 16035119

[ref21] Fattore L. Melis M. Fadda P. Fratta W. (2014). Sex differences in addictive disorders. Front. Neuroendocrinol. 35, 272–284. doi: 10.1016/j.yfrne.2014.04.003, PMID: 24769267

[ref22] Furnham A. (2022). “Bright and dark side of personality: the relationship between personality traits and personality disorders” in Overcoming bad leadership in organizations. eds. Lusk D. Hayes T. L. (New York: Oxford University Press), 51–75.

[ref23] Grunfeld R. Zangeneh M. Grunfeld A. (2004). Stigmatization dialogue: deconstruction and content analysis. Int. J. Ment. Heal. Addict. 1, 1–14.

[ref24] Jiménez-Murcia S. Granero R. Fernández-Aranda F. Stinchfield R. Tremblay J. Steward T.. (2019). Phenotypes in gambling disorder using sociodemographic and clinical clustering analysis: an unidentified new subtype? *Frontiers*. Psychiatry 10:173. doi: 10.3389/fpsyt.2019.00173, PMID: 30984045 PMC6450083

[ref25] John O. P. Srivastava S. (1999). “The big five trait taxonomy: history, measurement, and theoretical perspectives” in Handbook of personality: Theory and research. eds. Pervin L. A. John O. P.. 2nd ed (New York: Guilford Press), 102–138.

[ref26] Kaur P. Leino T. Chegeni R. Erevik E. K. Mentzoni R. A. Pallesen S. (2023). Association between problem gambling and personality traits: a longitudinal study among the general Norwegian population. Front. Psychol. 14:1241365. doi: 10.3389/fpsyg.2023.1241365, PMID: 38094699 PMC10716227

[ref27] Kessler R. C. Hwang I. LaBrie R. Petukhova M. Sampson N. A. Winters K. C.. (2008). DSM-IV pathological gambling in the National Comorbidity Survey Replication. Psychol. Med. 38, 1351–1360. doi: 10.1017/S0033291708002900, PMID: 18257941 PMC2293303

[ref28] Kruedelbach N. Walker H. I. Chapman H. A. Haro G. Mateu C. Leal C. (2006). Comorbidity on disorders with loss of impulse-control: pathological gambling, addictions and personality disorders. Actas Esp. Psiquiatr. 34, 76–82. 16552635

[ref29] Kurita S. Doi T. Tsutsumimoto K. Nakakubo S. Ishii H. Kiuchi Y.. (2021). Predictivity of international physical activity questionnaire short form for 5-year incident disability among Japanese older adults. J. Phys. Act. Health 18, 1231–1235. doi: 10.1123/jpah.2021-0247, PMID: 34433703

[ref30] Kwok C. Leung P. Y. Poon K. Y. Fung X. C. C. (2021). The effects of internet gaming and social media use on physical activity, sleep, quality of life, and academic performance among university students in Hong Kong: a preliminary study. Asian J. Soc. Health Behav. 4, 36–44. doi: 10.4103/shb.shb_81_20

[ref31] Langham E. Thorne H. Browne M. Donaldson P. Rose J. Rockloff M. (2015). Understanding gambling related harm: a proposed definition, conceptual framework, and taxonomy of harms. BMC Public Health 16, 1–23. doi: 10.1186/s12889-016-2747-0, PMID: 26818137 PMC4728872

[ref32] MacLaren V. V. Fugelsang J. A. Harrigan K. A. Dixon M. J. (2011). The personality of pathological gamblers: a meta-analysis. Clin. Psychol. Rev. 31, 1057–1067. doi: 10.1016/j.cpr.2011.02.002 21802620

[ref33] Miller L. Mide M. Arvidson E. Söderpalm Gordh A. (2023). Clinical differences between men and women in a Swedish treatment-seeking population with gambling disorder. Front. Psych. 13:1054236. doi: 10.3389/fpsyt.2022.1054236, PMID: 36684005 PMC9847389

[ref34] Millon T. (2006). Millon clinical multiaxial inventory–III (MCMI–III) manual. Third Edn. Minneapolis, MN: Pearson Assessments.

[ref35] Moreira D. Azeredo A. Dias P. (2023). Risk factors for gambling disorder: a systematic review. J. Gambl. Stud. 39, 483–511. doi: 10.1007/s10899-023-10195-1, PMID: 36884150 PMC9994414

[ref36] Myrseth H. Pallesen S. Molde H. Havik O. E. Notelaers G. (2016). Psychopathology and personality characteristics in pathological gamblers: identifying subgroups of gamblers. J. Gambling Issues 2016:68. doi: 10.4309/jgi.2016.32.5

[ref37] Okechukwu C. (2019). Role of exercise in the treatment of gambling disorder. Nigerian J. Exp. Clin. Biosci. 7:50. doi: 10.4103/njecp.njecp_11_19

[ref38] Oliveira J. M. Spositon T. Rugila D. F. Pitta F. Furlanetto K. C. (2023). Validity of the international physical activity questionnaire (short form) in adults with asthma. PLoS One 18:e0282137. doi: 10.1371/journal.pone.0282137, PMID: 36827240 PMC9956041

[ref39] Ortiz-Hernández L. Ramos-Ibáñez N. (2010). Sociodemographic factors associated with physical activity in Mexican adults. Public Health Nutr. 13, 1131–1138. doi: 10.1017/S1368980010000261 20196912

[ref40] Potenza M. N. Balodis I. M. Derevensky J. Grant J. E. Petry N. M. Verdejo-Garcia A.. (2019). Gambling disorder. Nat. Rev. Dis. Primers 5:51. doi: 10.1038/s41572-019-0099-7 31346179

[ref41] Rhodes R. E. Boudreau P. (2017). “Physical activity and personality traits” in Oxford research encyclopedia of psychology (Oxford University Press).

[ref42] Rhodes R. E. Smith N. E. I. (2006). Personality correlates of physical activity: a review and meta-analysis. Br. J. Sports Med. 40, 958–965. doi: 10.1136/bjsm.2006.028860, PMID: 17124108 PMC2577457

[ref43] Rocklin T. Revelle W. (1981). The measurement of extroversion: a comparison of the Eysenck personality inventory and the Eysenck personality questionnaire. Br. J. Soc. Psychol. 20, 279–284. doi: 10.1111/j.2044-8309.1981.tb00498.x

[ref44] Sáez-Abad C. Bertolín-Guillén J. M. (2008). Personality traits and disorders in pathological gamblers versus Normal controls. J. Addict. Dis. 27, 33–40. doi: 10.1300/J069v27n01_04, PMID: 18551886

[ref45] Tang C. S. K. Gan K. Q. Lui W. K. (2023). The associations between obsessive compulsive personality traits, self-efficacy, and exercise addiction. Behav. Sci. 13:857. doi: 10.3390/bs13100857, PMID: 37887507 PMC10603988

[ref46] Welte J. W. Barnes G. M. Tidwell M.-C. O. Hoffman J. H. Wieczorek W. F. (2015). Gambling and problem gambling in the United States: changes between 1999 and 2013. J. Gambl. Stud. 31, 695–715. doi: 10.1007/s10899-014-9471-4, PMID: 24880744 PMC4250449

[ref47] Wilson C. Butler N. Quigg Z. (2024). Harms from other People’s gambling: associations with an Individual’s own gambling Behaviours, health risk Behaviours, financial problems, general health, and mental wellbeing. J. Gambl. Stud. 40, 1–15. doi: 10.1007/s10899-024-10291-w, PMID: 38489134 PMC11390759

[ref48] Wong G. Zane N. Saw A. Chan A. K. K. (2013). Examining gender differences for gambling engagement and gambling problems among emerging adults. J. Gambl. Stud. 29, 171–189. doi: 10.1007/s10899-012-9305-1 22585283 PMC4736715

[ref49] World Health Organization (2010). “Global recommendations on physical activity for health” in Physical activity for health. 2nd ed. Geneva: World Health Organization.

[ref50] Wullinger P. M. Bickl A. M. Loy J. K. Kraus L. Schwarzkopf L. (2023). Longitudinal associations between psychiatric comorbidity and the severity of gambling disorder: results from a 36-month follow-up study of clients in Bavarian outpatient addiction care. J. Behav. Addict. 12, 535–546. doi: 10.1556/2006.2023.00026, PMID: 37307216 PMC10316174

[ref51] Xuan Y.-H. Li S. Tao R. Chen J. Rao L.-L. Wang X. T.. (2017). Genetic and environmental influences on gambling: a meta-analysis of twin studies. Front. Psychol. 8:281614. doi: 10.3389/fpsyg.2017.02121 PMC5723410 29259572

[ref52] Yamada M. Sekine M. Tatsuse T. (2023). Pathological gaming and its association with lifestyle, irritability, and school and family environments among Japanese elementary school children. J. Epidemiol. 33, 335–341. doi: 10.2188/jea.je20210365, PMID: 34744101 PMC10257987

