# Supplementary material for: Personality traits and physical activity in patients with gambling disorder attending a rehabilitation center. An observational study
Source: Front Psychol. 2024 Nov 12;15:1465195. doi: 10.3389/fpsyg.2024.1465195 (PMC11588469; doi:10.3389/fpsyg.2024.1465195)
Supplement: Supplementary file 1 [file Data_Sheet_1.PDF]

CUESTIONARIO INTERNACIONAL DE ACTIVIDAD FISICA

IPAQ: FORMATO CORTO AUTOADMINISTRADO DE LOS ULTIMOS 7 DIAS

PARA SER UTILIZADO CON ADULTOS (15- 69 años)

Las preguntas se referirán al tiempo que usted destinó a estar físicamente activo en los **últimos 7 días**. Por favor responda a cada pregunta aún si no se considera una persona activa. Por favor, piense acerca de las actividades que realiza en su trabajo, como parte de sus tareas en el hogar o en el jardín, moviéndose de un lugar a otro, o en su tiempo libre para la recreación, el ejercicio o el deporte.

Piense en todas las actividades ***intensas*** *que usted realizó en los* ***últimos 7 días.*** *Las actividades físicas* ***intensas*** *se refieren a aquellas que implican un esfuerzo físico intenso y que lo hacen respirar mucho más intensamente que lo normal. Piense solo en aquellas actividades físicas que realizó durante por lo menos* ***10 minutos*** *seguidos.*

1. Durante los **últimos 7 días**, ¿en cuantos realizó actividades físicas **intensas** tales como levantar pesos pesados, cavar, hacer ejercicios aeróbicos o andar rápido en bicicleta?

   ______ **días por semana**

   ☐ Ninguna actividad física intensa → ***Vaya a la pregunta 3***

2. Habitualmente, ¿cuánto tiempo en total dedicó a una actividad física **intensa** en uno de esos días?

   ______ **horas por día**

   ______ **minutos por día**

   ☐ No sabe/No está seguro

   ☐

Piense en todas las actividades ***moderadas*** *que usted realizó en los* ***últimos 7 días****. Las actividades* ***moderadas*** *son aquellas que requieren un esfuerzo físico moderado que lo hace respirar algo más intensamente que lo normal. Piense solo en aquellas actividades físicas que realizó durante por lo menos* ***10 minutos*** *seguidos.*

3. Durante los **últimos 7 días**, ¿en cuántos días hizo actividades físicas **moderadas** como transportar pesos livianos, andar en bicicleta a velocidad regular o jugar dobles de tenis? **No** incluya caminar.

   ______ **días por semana**

   ☐ Ninguna actividad física moderada → ***Vaya a la pregunta 5***

4. Habitualmente, ¿cuánto tiempo en total dedicó a una actividad física **moderada** en uno de esos días?

   ______ **horas por día**

   ______ **minutos por día**

   ☐ No sabe/No está seguro

*Piense en el tiempo que usted dedicó a **caminar** en los **últimos 7 días**. Esto incluye caminar en el trabajo o en la casa, para trasladarse de un lugar a otro, o cualquier otra caminata que usted podría hacer solamente para la recreación, el deporte, el ejercicio o el ocio.*

5. Durante los **últimos 7 días**, ¿En cuántos **caminó** por lo menos **10 minutos** seguidos?

   ______ **días por semana**

   ☐ Ninguna caminata → ***Vaya a la pregunta 7***

6. Habitualmente, ¿cuánto tiempo en total dedicó a caminar en uno de esos días?

   ______ **horas por día**

   ______ **minutos por día**

   ☐ No sabe/No está seguro

*La última pregunta es acerca del tiempo que pasó usted **sentado** durante los días hábiles de los **últimos 7 días**. Esto incluye el tiempo dedicado al trabajo, en la casa, en una clase, y durante el tiempo libre. Puede incluir el tiempo que pasó sentado ante un escritorio, visitando amigos, leyendo, viajando en ómnibus, o sentado o recostado mirando la televisión.*

7. Durante los **últimos 7 días** ¿cuánto tiempo pasó **sentado** durante un **día hábil**?

   ______ **horas por día**

   ______ **minutos por día**

   ☐ No sabe/No está seguro

RESULTADO EN NIVEL DE ACTIVIDAD

NIVEL ALTO (Actividad física vigorosa)
NIVEL MODERADO (Actividad física moderada)
NIVEL BAJO O INACTIVO

VALOR DEL TEST:

1. Caminatas: 3'3 MET* x minutos de caminata x días por semana (Ej. 3'3 x 30 minutos x 5 días = 495 MET)
2. Actividad Física Moderada: 4 MET* X minutos x días por semana
3. Actividad Física Vigorosa: 8 MET* X minutos x días por semana

A continuación sume los tres valores obtenidos:

Total = caminata + actividad física moderada + actividad física vigorosa

CRITERIOS DE CLASIFICACIÓN:

- Actividad Física Moderada:
    1. 3 o más días de actividad física vigorosa por lo menos 20 minutos por día.
    2. 5 o más días de actividad física moderada y/o caminata al menos 30 minutos por día.
    3. 5 o más días de cualquiera de las combinaciones de ca minata, actividad física moderada o vigorosa logrando como mínimo un total de 600 MET*.
- Actividad Física Vigorosa:
    1. Actividad Física Vigorosa por lo menos 3 días por semana logrando un total de al menos 1500 MET*.
    2. 7 días de cualquier combinación de caminata, con actividad física moderada y/o actividad física vigorosa, logrando un total de al menos 3000 MET*.

*** Unidad de medida del test.**
